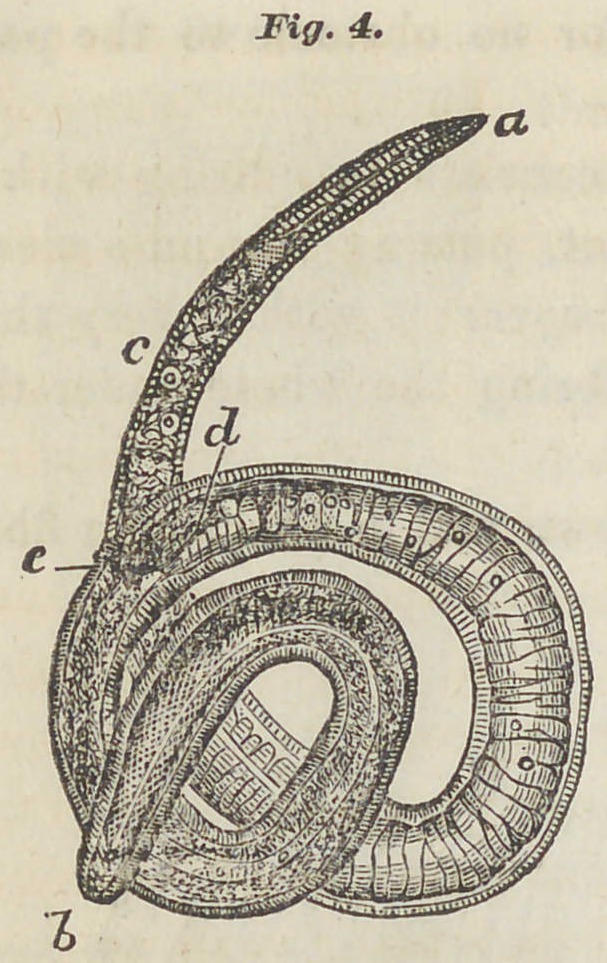# The Life of the Trichina

**Published:** 1868-11

**Authors:** Rudolph Virchow, Rufus King Browne

**Affiliations:** Prof. University of Berlin


### Original Communications.

### THE LIFE OF THE TRICHINA.

BY RUDOLPH VIRCHOW, M.D.PH.D., PROF. UNIVERSITY OF BERLIN.

Translated by Rufus King Browne, M. D.

[Continued from page 412.]

Recurring to the Trichina, if we wish to determine their presence, the question confronts us, where shall we investigate, *i. e.* from what part of the infested body is the meat for examination to be taken? Even in cases of slight infestation by Trichina, it is not of serious consequence what part is thus selected from, for they are generally found in all the muscles from the smallest to the largest; in those of the trunk, as well as those of the head and members. One set of muscular fibres alone is an exception, viz: the heart. Therefore, the eating of this last can be done without danger. But if the Trichina are spread through all the muscles, yet they seem to be more numerous in some than in others. These are the ends of the muscles, *i. e.* those portions where the muscle adjoins the tendons and the bone.

*Fig. 2.*

In the adjoining figure, in a part of the muscles forming the calf of the human leg, is shown the aggregation of the Trichina around the beginning of the tendon. The white light striped places show the tendon, the dark, denser stripes the muscle. Around the latter, near the end of the muscle, is seen a garland of the Trichina capsules.

This peculiar appearance may be explained by the fact that the Trichina in their migration proceed toward the end of the muscles, to a point where their further progress is stayed by certain obstacles. For the diagnosis of the disease in man, the practical deduction is, that if we wish to select a part of the muscle for special investigation it should be from the vicinity of a tendon.

All we have said hitherto relates only to the encapsuled Trichina, which are already encased in lime. But how shall we find those which are not in this state, or are in the process of becoming encapsuled?

Without the microscope this is altogether impossible. To be sure I have seen with the naked eye a full grown free Trichina, as a white point, but whether this point was an animal I could not with certainty affirm.

The movements of a free Trichina are scarcely recognizable. It never amounts to a change of posture of its entire body. In unusual conditions it moves slightly, but usually this consists of mere diminution and return of the breadth of the coil.

These changes of movement are so very slight that they are quite invisible to the naked eye. But if the animal be wholly uncoiled it will be invisible, since the very narrow transparent body interposes little or no obstacle to the perfect transmission of light.

The best mode of proceeding consists in cutting with a sharp knife a small bundle of meat, putting this on a clean glass plate, add a drop of water, cover it with a very thin slip of glass, pressed down, and bring the whole under the microscope.

Below, in cut *B*, a Trichina is shown within a muscular fibre

*Fig. 3.*

which has become enlarged by its presence. This piece is from the muscle of a person dead from too large a migration of Trichina (in the epidemic of Burg).

We now speak, not of capsules, but of the Trichina itself. We therefore describe the animal.

*Fig. 4.*

A full grown old muscle Trichina, as shown 300 times magnified in Fig. 4, is a round worm that is similar in shape to a rain-worm. It has an anterior pointed end *a*, in which is the mouth, from which a narrow tube, the æsophagus, proceeds. The æsophagus is surrounded by a thick body of cells *c*, which stretch through a great part of the body, and end in a fine intestine near *d*. The intestine runs to the posterior end where it terminates in an orifice.

Near *e* is seen a dark heap of granules. This is placed in the sexual canal which occupies a large portion of the posterior end of the body. These two main parts, the digestive and sexual, are covered by a strong external skin which shows fine transverse wrinkles.

The Trichina is, therefore, a fully organized animal of the class worms, the inner structure of which can be well recognized on account of the transparency of its skin. But of course this can only be done with a microscope, and a lens of 300 diameters. With inferior instruments, and low powers, one can see but little more than the outer shape of the worm. For common purposes this is sufficient, as well for the investigation of meat as the diagnosis of disease, and to preclude the possibility of an error in the case. It is to be remarked that no species of maggot has any resemblance to it, especially the larvæ of flies have quite another shape, and are a great deal larger, and if ignorant butchers and others assert that the Trichina affair was that of harmless maggots, it is only a sign of exceeding ignorance and levity.

Trichina are found in instances of flesh migration loose in the flesh, and if we cut out a small piece and lay it in a drop of water upon a glass plate, quite a number of them float around the piece of flesh. But the capsules hold similar animals in older cases, even those when the capsules have become chalky, and we can free them by a slight pressure.

When the chalking is incomplete, and the capsules have attained a certain tenacity and rigidity, they burst under slight pressure, and the animal is extruded. When one has taken the particles of meat, and placed them upon a glass plate, and covered them with a thin glass, to press upon the latter suffices to dislodge them from the capsules; this statement will make it clear that an understanding of the Trichina pre-supposes an application of the microscope, and that only in the fullest development, and the state of chalkiness of the capsules they can be seen with the naked eye.

What is the danger from the Trichina to the human body? In the historical part of this treatise we mentioned that more than two deceniums since the discovery of the Trichina have elapsed, and there was not attributed to them any dangerous action on the human body. It was also said that only cured cases had been observed, and even these had been rarely investigated. Years passed without a single new case becoming known, and even to this day, in France, only a single observation has been made; while in other countries not a single one has been published.

I have first shown that with careful observation a greater frequency of the presence of Trichina will be found. In the single year of 1859[1], six times I found the animals in human corpses, and in a short time I had seen more cases than had been reported in the whole literature of the world for thirty preceding years.

I may say, that last November I found four new cases of Trichina in persons who had died at the "Charity Hospital." Other investigators have found similar cases.

1. Virchow's Arch. XVIII, pp. 330.

One has to remark here that all these were first recognized by dissection, and while these persons lived no one had a misgiving of their condition. All the Trichina found in these cases were encapsuled, they were, therefore, all cured cases, but they have a weighty significance, as they show that the possibility of a danger we fear in other cases is not far removed.

But these experiences would not have been sufficient to arouse the general interest, if other cases of fresh migration, not encapsuled, and free Trichina had not been discovered, and if we had not been led by this to investigate the source of these migrations, and if, finally, no epidemic of cases, and cases of death in consequence thereof had occurred.

It is the merit of Zencker,[1] that he first, in and near Dresden, observed such an epidemic, and also showed in the ham and sausages made from one particular pig, the Trichina. This pig had been butchered on a farm near Dresden. The butcher, the owner of the farm, and other people had fallen sick, and a previously perfectly healthy servant girl had died. In her body an abundance of Trichina were found. I received from Mr. Zencker a piece of the ham, as well as a muscle of the girl, and, therefore, had the opportunity not only to verify his previous observations, but with this material to make a series of experiments on animals which I will shortly state:

A rabbit fed with Trichina from the girl, died in a month with its flesh full of them. Some of this flesh was given to a second rabbit. It also died in a month. With this meat three other rabbits were fed. Of these, two died at the end of three weeks, and the third in the fourth week. To another animal the meat of these was fed. As it ate but little it lived six weeks. In all of these the muscles after death were found filled with Trichina. Even in the smallest particle of their meat several were found.

---

1. Virchow Arch. XVIII, pp 561.

To be certain that this result was in reality due to the fed Trichina meat, I examined parts of the muscles of some of these animals before they were fed. I did not find a single Trichina, and they have never been found in these animals unless they had previously been fed with meat containing Trichina. A few weeks after feeding the muscles of the same animals which I had found free of them, were filled with them. Convincing as these experiments of the infection resulting in death were, through five generations of animals, one might still imagine them to be the result of chance.

To preclude this possibility, there was only to be shown that the migration and infestation really proceeded from the feeding of the animals with Trichina. This, however, could be demonstrated. It could be proved that the Trichina in the fed meat became free in the stomach and intestines of the rabbit, and become individuals of both sexes which in a short time attained a length of from three to four millemetres, and with the naked eye can be seen as fine white threads. Eggs are produced in the female, and from these, young even in the body of the mother, which about a week later are born, and move about freely in the mucus of the intestine. The Trichina are, therefore, animals which beget living young.

The young are exceedingly minute. They are the smallest of the class of thread-worms known. It is the young which leave the intestine and penetrate the body.

I have found them in the lymphatic glands of the mesentery, in the peritoneal cavity, and in the pericardium, and in the muscles. In the latter they find the suitable conditions for their further development. Here they increase, and in from three to five weeks attain the size of their parents at the time they were fed.

The above series of investigations which I communicated to the Paris Acad. of Sciences at their session of July 2d, 1860, allow no doubt of the history and importance of the Trichina. I have myself repeated these investigations several times, and other observers have done likewise. If we add to these the observations made on men which increase every year, it must be regarded as folly or criminal to speak of unfounded fear of Trichina (Trichinaphobie).

A whole series of cases, or as they are called epidemical, are well established. I refer to the epidemics of Plauen,[1] Calbe on Saale,[2] Quedlinburg,[3] Burg near Magdeburg, Weimar and Hettstadt near Eislaben, as well as the very curious case which happened on a Hamburg vessel.[4] Several other epidemics which have been probably caused by Trichina, I do not report because of there having been no microscopic investigation. In the other epidemics there were many cases. 20 to 50 persons were infested, but in the case of Hettstadt 150 persons fell sick, several of these very severely, and the number of deaths were above 20.[5] There is no room, therefore, for doubt. The most reliable observations have been made. I have examined flesh from the cases at Burg and Hettstadt, and found them literally filled with Trichina.

It is not the purpose of this treatise to dilate upon the symptoms of the disease in particular. It may suffice to say that the symptoms are various. Sometimes they are irritation of the intestines (intestinal catarrh,) dysentery, gastric disturbances, and muscular disease, as weakness, stiffness, pains, resembling gout or rheumatism, and a febrile condition similar to that of typhus fever. Sometimes the train of symptoms are acute and death occurs in the fourth or fifth week. Sometimes they proceed more slowly, and after weeks, convalescence occurs, with chronic disease, emaciation, and loss of strength. In several cases I have examined the dead bodies of those supposed to have been victims of consumption. In

---

1. Bähler the Trichina Disease, and Its Treatment in Plauen in 1863.
2. G. Simon, Prussian Med. Zeitung. 1862. No. 38 to 39.
3. Behrens Deutsche Klinick. 1863. No. XXX.
4. Tüngell, Virchow's Arch. 1863. Vol XXVII. pp 421.
5. Up to Nov. 23d, 137 cases of disease, and 24 cases of death had occurred.

these, with very little affection of the lungs I found many Trichina, with great decrease of muscular substance[1]

To the experienced physician, these cases of disease have peculiarities by which they are distinguished from gastric and nervous fevers, from gout and rheumatic affections, but a perfectly reliable judgement can only be formed if Trichina have been found in the meat which was eaten, or in the body of the patient himself, but the latter is only possible if a small piece of muscle be taken by an operation from the body of the patient. Without the certainty of the presence of the animals, we formerly were in doubt, as to the nature of the case, and the supposition of poisoning was prevalent.

Since the year 1860, I and several other observers have endeavored to spread a knowledge of these facts, and to call attention to the danger which might result from an incautious eating of pork. From the beginning, the opposition of the butchers was raised, and even now it still exists. I remark, however, that it is precisely these tradesmen who should be most interested in adopting every precaution, for the danger not only affects their trade, but their personal welfare.

In some of the epidemics, as well as in a series of single cases, for instance those of Friedrich,[1] Traube,[2] Tüngel, it was just the butchers who were infected by the pig they killed. Of course we have no idea of infection by the skin, but the butchers not merely eat of the sausages, but most of them are in the habit of eating a little of the uncooked meat, at the time of cutting it up, as well as that which adheres to the knife during that process. They, therefore, are the first exposed to the danger. Next came the cooks and servants, and lastly, the rest of the population. But even after the occurrence of the Trichina disease in human beings could not be doubted, they busied themselves in a most unconscientious manner to throw a doubt upon these facts. Uninformed and

---

1. Friedrich. Virchow's Arch. 1862. XXV. pp 399.
2. G. Schultze de Trichinäsi Diss. inaug. Beril. 1863. pp 17.

evil minded persons spread the assertion that the disease had not even been shown in the pig. This is utterly untrue.

As I said in a former portion of this paper, Leidy, in North America, 16 years ago, found Trichina in a pig. Zencker found them in the ham and sausages of the pig partaken of by the patients and servant girl who died in the Dresden epidemic. He sent me one piece of that ham, and I ascertained that it contained Trichina.

The same results have been found in the epidemics at Quedlinburg and Hettstadt, in hams in the former and in sausages in the latter. In Hettstadt it happened that most of the people who were taken ill had participated in a feast, which took place on the 10th of October of last year. Most convincing, however, is the case described by Tüngel.

The second argument is no more reasonable. The most careful trials with feeding, by Haubner and Kuchenmeister, and Leisering,[1]. pigs with Trichina-meat resulted in showing that some of these animals sickened and died, but in their conclusions they state that the pigs so fed, presented no peculiar symptoms, such as would be indicative of this disease alone, and furthermore, the reports of the cases of epidemics contain no mention of any such peculiar symptoms in the pigs whose meat caused the disease in human beings.[2] But, even if we admit that these peculiar symptoms of infection exist in the pig, these will not be apparent when the pigs are in market and for sale.

Such are cases of already cured and encapsuled Trichina.

---

1. Haubner, Kuchenmeister and Leisering Helminthdogie Investigations. Dresden. 1863. pp 5. (From the Report of the Veterinary Department of the Kingdom of Saxony for the year 1862.

2. Dr. Rupprecht, in Hettstadt, wrote me respecting the pig from which proceeded the infection there. It was a two and a half-year old mother pig, who appeared to five butchers perfectly healthy, another, and the sixth, bought it. It, therefore, did not appear suspicious to him, for he and seven members of the family ate the meat and became sick.

The real symptoms of disease may have appeared months before, and in single cases very honest owners offering them may acknowledge that the pigs had been sick. But there is no security in these circumstances. If one considers how many pigs come to market especially in large cities, not only from the distance of miles, but also from more distant provinces and countries, it is impossible to investigate the history of the previous state of health of the animals.

It has been shown that encapsulation and the infiltration of the capsule with chalk does not kill the animals. In most cases in human beings where I found these capsules, the enclosed animal was still alive. How long had elapsed since the migration I can not state, for in all these cases the time when they first entered the body could not be ascertained. But it is certain, that months must elapse before the deposit of carbonate of lime commences, and it is highly probable that the Trichina may remain in the body of the human subject in a state of suspended animation, to awake to new activity as soon as they are taken into another body. I have repeatedly, with success, fed such trichina to animals. Notwithstanding all argument, and the question is not merely allowed but enjoined, why is it that the accidents which follow the eating of Trichina-meat are so various in their degree and significance?

A ship from Hamburg returned from Valparaiso. Before quitting the captain purchased a live pig. It was slaughtered by the cook on board the ship, on the 1st of April, 1863. The crew ate 30 pounds of the pork fresh, and the remainder was salted. On entering the port most of the sailors fell sick, most of them slightly so, some, however, severely, and two died. In a 16 year old youth, who died on the 24th, numerous free living Trichina were found in the muscles. The salted pork, a piece of which I obtained and examined, contained Trichina though dead.

That Trichina are found in pigs, and that people who eat this meat fall sick, can no more be doubted. Most people are consoled with the idea that the infection of pigs is not frequent, and that those who have them must evince the symptoms. The first idea may be admitted, but what consolation does it afford those who have the misfortune to eat of the meat of the few pigs which have Trichina? This question is to be answered definitely.

The intestinal consequences (gastric fever, dysentery) are consequent upon the presence of the animals therein. The length of this period is indefinite. If after persons have eaten the meat they have severe diarrhea, it may be possible that all the animals are discharged. If not, they grow, move about, procreate, and then comes the morbid irritation; this appears differently in different individualities. Sensitive persons, who are liable to diarrhea are, on the whole more secure from infestation than persons inclined to costiveness.

The muscular and febrile symptoms proceed from the penetration of the young from the intestine into the body. But these too vary with individual peculiarities. Certain conditions of the intestine may favor their migration or otherwise.

I never succeeded in producing the disease in dogs, although the development of Trichina in their intestines is very complete, for the first I discovered were in a dog. We have, as yet, no statistics on the subject as the observations made do not furnish sufficient data. The second idea is still less valid.

If the migration is in course of progress, the danger is in the ratio of the number of migrating animals. This, however varies. I have seen cases of human bodies where I could only by a very extended search find about a dozen Trichina in the muscle. There are other cases of millions. The noticable effects are produced by the action of the animals on the places to which they penetrate.

A person who has but a dozen or so will probably never have any perception of it—his health will not be disturbed for a moment. A second, into whose muscles thousands have penetrated will have all kinds of disagreeable sensations, as pains in the muscles, stiffness, weakness, heaviness in his limbs, hoarseness, &c., but he will pass through these symptoms, the migrating animals will become encapsuled, and enclosed with lime. Thus a cure will be effected. A third, into whose muscles millions migrate will, perhaps, recover too, but very slowly. He will remain weak, diseased and very thin; or, he may not recover and may die by advancing disturbance of all muscular activity, especially that of respiration. This is evident if we remember the three cardinal points of the Trichina elucidation:

1. The eaten Trichina remain in the intestine, and never enter the muscles.

2. They produce living young, which enter the muscles.

3. The young which have entered the muscles grow there but do not multiply.

The main danger, therefore, is in the production of young by the intestinal Trichina.

A grown Trichina mother has a hundred of living young in her body, and after these young she always produces more and more eggs. How long she lives and produces young is not exactly known, but at least four to five weeks. It is certain that she is fixed in the intestine and continues to produce new broods of young. If we calculate that one Trichina mother has 200 young; twenty thousand such mothers are sufficient to produce a million of young, and so many female animals may be contained in a few morsels of meat.

Even if there is no high degree of infestion. A glance at a small piece of muscle shown in the engraving is sufficient to prove this calculation.

The more Trichina that are eaten, and the longer they remain in the intestine the more young will be produced and the danger increased. I have proved this directly by experiments on animals.

A rabbit which ate only a small part of meat containing few Trichina does not become diseased. In the epidemic of Burg this was strikingly illustrated. A woman who ate raw meat on bread died. Her child, which only licked the spoon with which she had spread the meat, was slightly sick.

A person, therefore, as well as a pig can have Trichina in his body, without causing very serious illness or death. That is, however, a small comfort inasmuch as an absolute preventative against Trichina can not be found, and inasmuch, moreover, as no special investigation of meat can be extended to all parts of it.

But it is certain that a very large migration of the animals into the human body produces disease and perhaps death; and this should suffice to suppress all objections raised against a careful investigation of the meat. Often it has been remarked that formerly one had not heard of such disease, and if the danger was so great it ought to have been discovered before. Specially such groups of cases could not have occurred without attracting notice.

Some persons have supposed that the disease must be a new one, and not have appeared. But this conjecture is always presented if a hitherto unknown disease has been recognized by careful investigation, and thus discriminated from a group of diseases of like character. I mention here a more formidable disease which can be transmitted from animals to man, the glanders. The first fully authenticated case of this disease has been reported by Schilling in 1821, and subsequently new cases are known every year. Should we therefore conclude that the glanders is a new disease? The glanders in animals has been mentioned by old Roman and Greek writers,[1] and we know not the least reason why they had not been transmitted to man. But it is difficult to prove that this or that case which has formerly been described was traceable to such a transmission.

It is well known that the Mosaic laws[2] denounced the pig as an unclean animal, and prohibited the eating of its meat. Perhaps this prohibition came from the observation that the

---

1. Virchow's Handbook of Special Pathologie and Therapie. Enlarged. 1365. Vol. II. pp 406–413.

2. Moses III. Chap. XI, Verses 7–8.

pig ate unclean and putrid food, but may we not suppose that at that time disease following the eating of pig's meat had occurred. In the more simple way of living of a people in a nomadic condition, the diseases which came in groups might easier be traced to its causes.

In discovering that the tape worm in man came from the eating of the cysticercus of pigs, it has been supposed that the Mosaic commandment was given on account of the danger from tapeworms. But tapeworms seldom cause real disease, and are not dangerous in the literal sense, and if the prohibition proceeded from a knowledge of a transmitted malady, the idea of the Trichina in connection therewith is more probable. Certainly most men do not sicken immediately after eating Trichina in meat. Days pass and suspicion may easily fall on a familiar cause. Still, if numerous persons get sick at the same time, the suspicion will finally be led to the true source.

There are in medical literature, especially in medical jurisprudence, a number of cases where suspicion was led to ham.

As Trichina were not known, and the muscles of the dead were not investigated in a judicial autopsy, this enquiry, otherwise very conscientious, did not give the clue. It poisoning was thought of, and in chemical analysis no mineral poison was found, one supposed an organic poison to exist and called it ham poison.

Does ham poison exist? No body can tell, for no chemist has ever been able to find it. The whole argument stops here—namely, that another poison can not be shown, and that poisoning does exist. But is the poisoning proved? No, it is only supposed because another explanation could not be given. The Trichina diseases give another explanation, and it may suffice to point out a few cases.

Last summer Herr Langenback operated on a man for swelled neck. During the operation the raw muscles were seen to be filled with Trichina surrounded with the chalk deposit. When it was enquired whether he had never been sick in a peculiar way, he told a strange story. In the year 1845 the members of a commission for inspection of schools in the Lausitz, at a meal, ate ham, sausage, cheese, etc. at an inn. One member partook of only some red wine. The other seven members drank white wine and ate of the provisions. All seven, in which number was included the relator, fell sick, and subseqently four died. Suspicion was aroused toward the inn-keeper, and the meal. Judicial investigation was had in which the white wine was tested, but without result; but the inn-keeper could not dispel the suspicion, and was eventually obliged to emigrate.

In June, 1851, in the neighborhood of Hamburg, a number of persons who had eaten ham afterward fell sick. Three died and several others were for a long lime in a low condition. The judicial investigation was here also without result, and finally, ham poison was supposed.

Parts of the ham remained, and its history could be traced to the butcher. It was then ascertained that the ham had been sold cheaper, on account of its poor quality, but what this inferiority of quality consisted in was not ascertained. Through the carefully conducted transactions in this case, Tüngel[1] proved that the symptoms and the cause were exactly identical with what we now know of the Trichina disease.

More cases might be named, but what is given suffices to prove that the disease has existed before we had any knowledge of Trichina, and the *need of the case is not the malady, but the knowledge of it.* Nobody, therefore, should try to hide the danger with such paltry reasoning, when only a conscious insight into the sources of the disturbance enables us to avoid, or in a great degree diminish it and its extent.

3. WHAT REMEDIES ARE THERE AGAINST TRICHINA DISEASE?

It is not our purpose to enter into technical details, but I will answer the question which has often been put to me, viz Is Trichina disease curable?

1. See Tüngel Virchow's. 1863. Vol. XXVIII. pp 391.

The foregoing remarks show that encapsulation is a kind of natural cure, for with the formation of the capsule the animal ceases its migration, and reposes in a sort of prison, where it leads such a torpid existence that its existence may be unindicated, but medical art can not hasten this process. It occurs in the natural course of proceedings, and can neither be promoted or hastened.

When the capsules have attained a certain degree of completeness, if the patient be alive the Trichina will not cause his death. It would be very desirable to have a remedy which would kill the Trichina, and not kill the infested person. It is imaginable to find such a remedy, for it is known that certain substances have a toxic effect on some animals, but not on others. But this remedy to destroy the Trichina has not yet been found. Arsenic, copper, mercury, phosphorus, camphor and spirits of turpentine have been supposed, but without any positive result. The picrio acid seemed to have had good results in a patient of Friedrich,[1] but this hope has been proved mistaken by Feidler and Moseler.[2] I will here remark, that it would be foolish to abandon scientific attempts at cure.

In attempting to find a cure, the first suggestion would be to aim at the muscle Trichina, because they are the dangerous guests, and to kill them would be the greatest gain. But the intestinal Trichina are also very dangerous. They produce the young which migrate, and upon the number of the intestinal Trichina and the period of their presence, depend the number of the muscle Trichina. Nothing, therefore, should be deemed more important than the getting rid of the mother animals which reside in the intestine and produce the young. If they are early removed no emigration of the young into the muscles occurs. If they are removed after the migration

1. Friedreich Virchow's Arch. XXV. pp 399.

2. Feidler " " XXVI. pp 573.

Moseler " " XXVII. pp 421.

has begun, it will then cease, and the danger not increase. The mother animals can only be expelled by vomiting and purging. Vomiting can only be resorted to soon after eating the affected meat while it is in the stomach. This will only be in instances in which the presence of the Trichina in meat, ham and sausage is early discovered. The usual mode must be purgation for the expulsion of the animals in this way is certain. In my communications to the Paris Academy in 1860, I mentioned that several rabbits fed at the same time with Trichina-meat, those that were purged were found free from Trichina. This has been confirmed by observations in men, and experiments with animals; and the practical rule is reached, that in cases where, probably or certainly, infection has taken place, we should resort to drastic purgatives.

Probably one will discover certain substances, which, similar to the case of the round-worm, will narcotize the Trichina, and such substances should be taken before the purgatives to facilitate *the expulsion* of the animals.[1]

These proceedings will not, however, benefit those patients in whose muscles a large migration has already taken place. But it is matter of great importance that we can aid these patients in whom only a very limited migration has taken place. In reference to this, those Trichina already in the muscles, I have no hope of finding a remedy to kill them. This has not even been done in the cysticercus, although we possess certain reliable remedies for tape worms. This is easily explained if we consider that all remedies which are designed to act upon these worms can only reach them through the blood. If a remedy is taken it has to be taken up by the blood, and carried to the muscles and enter them. Each remedy during this long progress is greatly diluted and

1. The recently published observations of Feidler Arch. Med. Knowledge. 1864. V. pp 21. (The purgatives applied to Trichinized cats and rabbits were without result, but these should not deter other experimenters from further trials.

reaches the particular muscle in very small and ineffectual doses. If the animals are already encapsuled they are least affected by the small particles of medicine. For the capsules being almost impervious presents an obstacle to the entrance of the substances. In my communication to the French Academy, I stated that I put Trichina-meat in a solution of chromic acid to harden it for microscopical research, and that in this solution, which was so strong that all the other parts had coagulated, so that it could be cut in very thin slices, the Trichina were fully alive. This was a case where the encapsulation of the Trichina was in an early stage.

## Figures and Tables

**Fig.2. f1:**
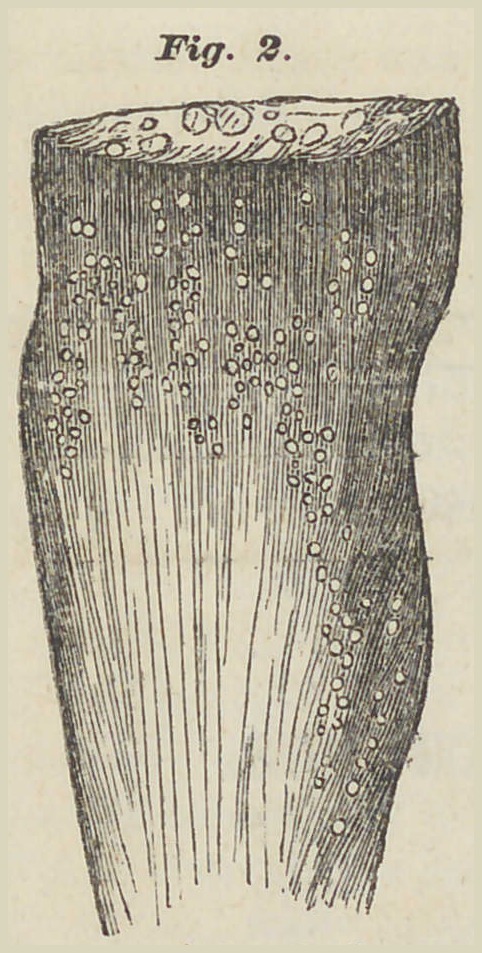


**Fig.3. f2:**
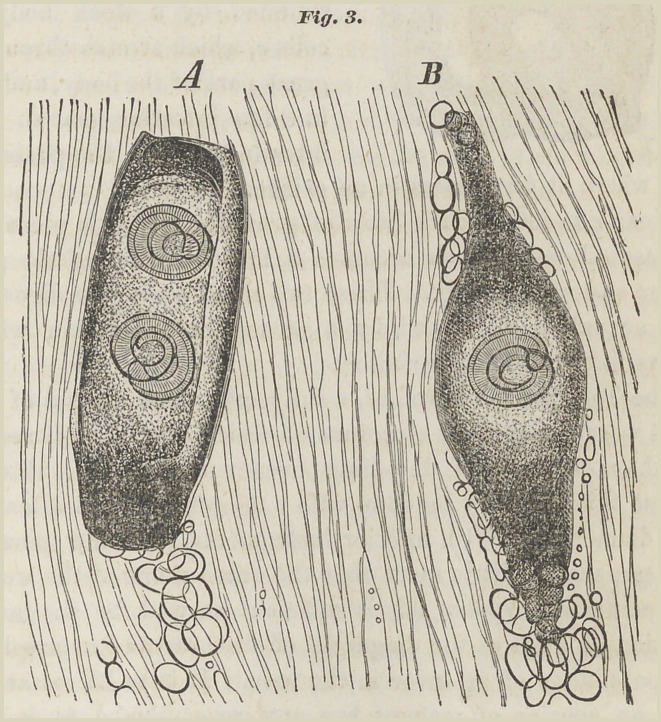


**Fig.4. f3:**